# Long-Read Spatial Transcriptomics of Patient-Derived Clear Cell Renal Cell Carcinoma Organoids Identifies Heterogeneity and Transcriptional Remodelling Following NUC-7738 Treatment

**DOI:** 10.3390/cancers18020254

**Published:** 2026-01-14

**Authors:** Hazem Abdullah, Ying Zhang, Kathryn Kirkwood, Alexander Laird, Peter Mullen, David J. Harrison, Mustafa Elshani

**Affiliations:** 1 School of Medicine, University of St Andrews, North Haugh, St Andrews KY16 9TF, UK; ha82@st-andrews.ac.uk (H.A.); yz217@st-andrews.ac.uk (Y.Z.); pm72@st-andrews.ac.uk (P.M.); djh20@st-andrews.ac.uk (D.J.H.); 2 Pathology, Western General Hospital, Crewe Road South, Edinburgh EH4 2XU, UK; kathryn.kirkwood@nhslothian.scot.nhs.uk; 3 Edinburgh Urological Cancer Group, Institute of Genetics and Molecular Medicine, Western General Hospital, Crewe Road South, Edinburgh EH4 2XU, UK; alexander.laird@nhs.scot; 4 NuCana plc, 3, Lochside Way, Edinburgh EH12 9DT, UK

**Keywords:** clear cell renal cell carcinoma, patient-derived organoids, organoid spatial transcriptomics, NUC-7738, heterogeneity, long read sequencing, transcript isoforms

## Abstract

Background: Clear cell renal cell carcinoma (ccRCC) is the most common subtype of kidney cancer and is marked by pronounced intra-tumoural heterogeneity that complicates therapeutic response. Patient-derived organoids offer a physiologically relevant model to capture this diversity and evaluate treatment effects. When integrated with spatial transcriptomics, they might enable the mapping of spatially resolved transcriptional and isoform-level changes within the tumour microenvironment. Methods: We established a robust workflow for generating patient-derived ccRCC organoids, that are not passaged and retain original cellular components. These retain key features of the original tumours, including cancer cell, stromal, and immune components. Results: Spatial transcriptomic profiling revealed multiple transcriptionally distinct regions within and across organoids, reflecting the intrinsic heterogeneity of ccRCC. Isoform-level analysis identified spatially variable expression of glutaminase (GLS) isoforms, with heterogeneous distributions of both the GAC and KGA variants. Treatment with NUC-7738, a phosphoramidate derivative of 3′-deoxyadenosine, induced marked transcriptional remodelling of organoids, including alterations in ribosomal and mitochondrial gene expression. Conclusions: This study demonstrates that combining long-read spatial transcriptomics with patient-derived organoid models provides a powerful and scalable approach for dissecting gene and isoform-level heterogeneity in ccRCC and for elucidating spatially resolved transcriptional responses to novel therapeutics.

## Simple Summary

Clear cell renal cell carcinoma is the most common form of kidney cancer. Recent advances in spatial and long-read sequencing now allow detailed examination of gene activity within tumours. We used non-passaged, patient-derived tumour organoid models which preserve the structure and cellular composition of the original tumours. Long-read spatial transcriptomics was then applied to study gene expression and transcript variants across organoid regions. We also examined how these patterns change following treatment with the experimental medicine NUC-7738. There are distinct spatial patterns in genes related to protein synthesis and energy metabolism, as well as region-specific differences in transcript isoforms. This work provides new insight into the molecular diversity of kidney cancer and demonstrates how advanced sequencing technologies can be used to study treatment effects in physiologically relevant tumour models.

## 1. Introduction

Renal cell carcinoma (RCC) is a major health burden, with 434,419 new cases reported globally in 2022, resulting in 155,702 deaths [1]. Clear cell renal cell carcinoma (ccRCC) is the predominant histopathological subtype, accounting for approximately 70% of all cases [2]. Drug response in RCC is highly variable due to extensive intra- and inter-tumoural heterogeneity, making it an ongoing challenge to predict which patients will benefit from specific treatments [3,4]. While targeted therapies have been developed and approved in recent years, the effectiveness of these drugs remains inconsistent, highlighting the need for better strategies to identify actionable targets related to tumour heterogeneity and its microenvironment [5].

The development of precision medicine for RCC has been limited by the dearth of reliable preclinical models that faithfully recapitulate the genetic and structural complexity of patient tumours. Conventional cell lines and patient-derived xenograft (PDX) models each have significant shortcomings in preserving the tumour’s native architecture and microenvironment [6]. Using primary tissue to generate organoids provides a promising alternative. However, there is little work published for ccRCC, and organoids that have been passaged lack elements of tumour microenvironment, including stromal and immune components [7]. In the present study, we used patient-derived ccRCC organoids (PDOs), which preserve stromal and immune elements as well as the heterogeneity of the original tumour [8]. A recent study compared the mutational landscape and mRNA expression profiles of renal organoids [9] but did not include spatial analysis of gene expression.

Understanding the complexity of gene expression at the transcript level is crucial, as alternative splicing increases transcriptome complexity and plays essential roles in tumour development and progression [10,11]. Spatial transcriptomics technologies, including the 10× Genomics Visium platform, have enabled high-throughput spatial mapping of gene expression in tumour tissues by capturing poly-adenylated RNA on spatially barcoded slides [12,13,14]. However, these methods predominantly rely on short-read sequencing, which capture 3′ tags and are then subsequently digested to smaller fragments, thus missing critical information about full-length transcript variation.

In this proof-of-principle study, we used patient-derived ccRCC organoids established from primary tissue without passaging [8]. Organoids were sectioned onto 10× Genomics Visium slides for spatial transcriptomic profiling, and barcoded libraries were generated using Oxford Nanopore Technologies Chemistry v14 to enable long-read sequencing of gene and transcript isoform expression. In addition to the spatial characterization of untreated organoids, we examined the effects of NUC-7738, a phosphoramidate derivative of 3′-deoxyadenosine currently in clinical evaluation, with further details available in Schwenzer et al. [15].

## 2. Materials and Methods

### 2.1. PDOs Culture

The study was conducted in accordance with the Declaration of Helsinki and approved by NHS Research Scotland Lothian Bioresource (20/ES/0061; SR1934). Informed consent was obtained from all patients for the use of their tissue in research. Clear cell renal cell carcinoma (ccRCC) biopsies were collected from patients undergoing partial or complete nephrectomy at the Western General Hospital, Edinburgh, and transported in Advanced DMEM/F12 medium #12634-010 (Gibco, Paisley, UK) supplemented with 10 mM HEPES #15630-056 (Gibco, Paisley, UK), 2 mM GlutaMAX #35050-061 (Gibco, Paisley, UK), and 1% antibiotic–antimycotic #A5955 (Sigma, Paisley, UK) at 4 °C. Tumour tissue was minced into 2 mm fragments; a portion was fixed in 10% formalin for histology, while the remainder was digested in DMEM/F12 containing 5 mg/mL Collagenase Type II #17101-015 (Gibco, Paisley, UK) and 10 µM ROCK inhibitor Y-27632 #1254 (Tocris Bioscience, Bristol, UK,) at 37 °C for 30–60 min. The digested material was filtered through a 70 µm cell strainer #352350 (Corning Inc., Corning, NY, USA) to obtain a single-cell suspension, treated with red blood cell lysis buffer #12770000 (Invitrogen, Invitrogen, Paisley, UK), washed, and counted. Approximately 5 × 10^6^ cells were seeded into 100 mL spinner flasks containing 45 mL Advanced DMEM/F12, 5 mL fetal calf serum (10%), 10 µM ROCK inhibitor, and 50 ng/mL human recombinant EGF #PHG0315 (Gibco, #12770000). Cultures were maintained at 27.5 rpm and 37 °C with 5% CO_2_ for 21 days prior to treatment. A subset of organoids was treated with 30 µM NUC-7738 for 24 h, while DMSO-treated organoids served as controls. Following treatment, six NUC-7738-treated and eight control organoids were processed for Visium spatial transcriptomics.

### 2.2. 10× Genomics Visium Experiments

The Visium Spatial Tissue Optimization Slide & Reagent Kit (10× Genomics, Pleasanton, CA, USA) was used to optimize permeabilization conditions for OCT-embedded ccRCC organoids, including determining the optimal cycle number for the amplification step, which is critical for achieving optimal library yield. Following this, spatially barcoded full-length cDNA was generated using the Visium Spatial Gene Expression Slide & Reagent Kit (10× Genomics, Pleasanton, CA, USA) according to the manufacturer’s instructions.

### 2.3. Image Acquisition

After methanol fixation and H&E staining, the Visium slide was scanned at 20× magnification using the Zeiss Axio Scan Z1 (Zeiss, Oberkochen, Germany) with a custom scanning profile that includes tissue focusing and the capture of fiducial marks. The acquired images were saved in .czi file format. Each slide section was then exported as a high-resolution bigTIFF file for downstream analysis.

### 2.4. Long-Read Nanopore Sequencing

After amplification, 10 ng of barcoded spatial cDNA transcripts were used for Nanopore library construction following the manufacturer’s protocol with some modifications. Briefly, the 10× Genomics Visium amplicons were tagged with biotin using the primers:

[Btn]Fwd_3580_partial_read1

(5′-/5Biosg/CAGCACTTGCCTGTCGCTCTATCTTCCTACACGACGCTCTTCCGATCT-3′)

and Rev_PR_partial_TSO_defined

(5′-CAGCTTTCTGTTGGTGCTGATATTGCAAGCAGTGGTATCAACGCAGAG-3′).

After annealing and amplification of the biotinylated oligonucleotides, the transcripts were captured using M280 streptavidin beads (Invitrogen, 11205D). The captured transcripts were further annealed and amplified using the cPRM primers from the SQK-PCS114 kit (Oxford Nanopore Technologies, Oxford, UK), followed by ligation of the Nanopore adapters. The prepared library was then loaded onto R10.4.1 PromethION flow cells and sequenced using the P2 Solo sequencer (Oxford Nanopore Technologies, Oxford, UK).

### 2.5. Long-Read Nanopore Data Processing

Nanopore libraries were sequenced using MinKnow software v24.06.8 (Oxford Nanopore Technologies, Oxford, UK) with default settings, and raw reads were saved in .pod5 file format. Basecalling of the raw reads was performed using the dorado super accurate (SUP) model (v0.7.0) on four Nvidia A100 GPUs (NVIDIA, Santa Clara, CA, USA), with parameters set to --minscore 7, --barcode-both-ends, and --no-trim. The unaligned .bam files generated by dorado were used as input for the epi2me-labs/wf-single-cell (v2.2.0) pipeline, utilizing the 2024 10× Genomics reference genome and the parameter --kit ‘visium:v1’. This workflow extracts cell barcodes and UMIs from 10× Genomics libraries, producing genome aligned tagged .bam files and generating gene and transcript count matrices for downstream analysis.

### 2.6. Spatial Analysis

In order to obtain the 10× Genomics Visium spatial barcode positions, a custom BAM-to-FASTQ script, bam2fastq.sh, was generated. Briefly, the script used the tagged.bam file containing demultiplexed reads from epi2me-labs/wf-single-cell and generated FASTQ files compatible with 10× Space Ranger. The spaceranger count function was then used to analyze the FASTQ files, from which we obtained the tissue_positions.csv file. Prior to Giotto object construction, barcode alignment between Visium spatial coordinates (tissue_positions.csv) and ONT expression matrices was validated by confirming the intersection of barcodes from each source; only spots with matched barcodes in both datasets were retained for downstream analysis. Raw gene and transcript expression matrices generated by epi2me-labs/wf-single-cell were subsequently processed using the Giotto Suite package (version 4.1.5) [16].

Within Giotto, spots were filtered based on defined thresholds, including spots “in_tissue”, a minimum expression threshold of 1, feature detection in at least 30 spots, and a minimum of 550 detected features per spot. A scree plot was then used to assess the number of principal components to retain for dimensionality reduction; Leiden clustering was performed using doLeidenCluster (resolution = 1.20) on a shared nearest neighbour (sNN) network (n_iterations = 1000). Plots visualizing the results were generated using Giotto’s plotting functions. Gene and transcript-level differentials were identified using the build-in scran method [17] for differential expression analysis via findMarkers_one_vs_all (min_feats = 10) using the default Holm-corrected *p*-values.

### 2.7. Differential Isoform Detection

Differential transcript usage (DTU) across Leiden clusters, organoids, and treatment conditions were identified using a two-step approach. First, the transcript-level outputs from epi2me-labs/wf-single-cell were used to construct a Giotto object. Within Giotto, data were normalized (scale factor = 6000 and spatially annotated based on cell/spot coordinates to assign them to specific Leiden clusters or Visium sections. This provided a spatially informed transcript count matrix annotated with corresponding transcript ids. Next, these annotated counts were analyzed with DRIMSeq [18], a statistical tool designed to detect DTU using default analysis with Benjamini–Hochberg adjusted *p*-values. A design matrix reflecting the experimental conditions (e.g., cluster identity, treatment status, or other spatial categories) was used. By fitting models to these conditions, DRIMSeq tested for changes in isoform proportions.

## 3. Results

To demonstrate the feasibility of utilizing spatial transcriptomics and long-read sequencing in ccRCC, we used organoids that maintained key histological features of the tumour microenvironment. We sectioned organoids, each measuring less than 2 mm in diameter, onto 10× Genomics Visium slides, followed by on-slide reverse transcription and PCR amplification and image capture, yielding sufficient barcoded transcripts for the construction of a Nanopore Chemistry v14 library. From the Visium–ONT sequencing, we obtained ~104 M and ~105 M raw reads for the DMSO control and 30 µM NUC-7738-treated sections, respectively. After non-full-length filtering with the epi2me-labs/wf-single-cell pipeline, 80 M reads (DMSO) and 65 M reads (NUC-7738) passed QC, with a mean read length of 800 bp. Following spot filtering in Giotto using the filterCombinations function, based on a minimum detected features per cell, 154 spots (DMSO) and 143 spots (NUC-7738) remained for downstream analysis. For completeness, whole-section views showing the full tissue context of Leiden clustering and UMAP-based spatial distributions, are presented in Supplementary Figures S1–S6.

### 3.1. Spatially Resolved Gene Expression Analysis in Control ccRCC Organoids

After Nanopore raw reads were basecalled using Dorado, key gene and transcript-level matrices were generated using the wf-single-cell pipeline. This process demultiplexed spatial barcode information and mapped reads to the reference genome, enabling downstream analysis. Resulting output of gene and transcript-level matrices were used to create a Giotto object [16], and data were filtered, normalized, and Leiden clustering applied. These clusters represent distinct gene expression profiles, of which five were identified. Supplementary Table S1 lists the top-ranked differentially expressed genes for each Leiden cluster, including raw *p*-values, FDR-adjusted *p*-value log fold change, cluster identity, and ranking. Their spatial localization highlights the inter-organoid heterogeneity even though the organoids were derived from the same patient.

**Figure 1:**
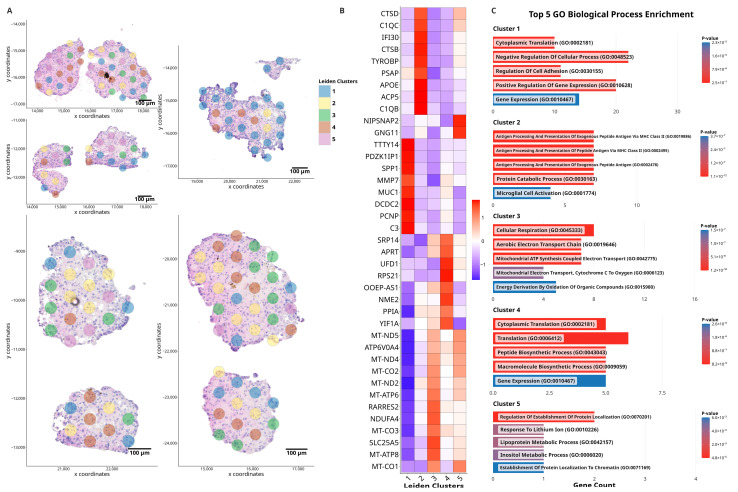
Spatial clustering, gene expression, and pathway enrichment analysis of section of untreated control ccRCC organoids. (**A**) Spatial distribution of 10× Genomics Visium spots overlaid on the H&E-stained image of organoids. Each spot represents spatial transcriptomic data, and the colour-coding indicates distinct gene expression clusters identified through unsupervised Leiden clustering, highlighting regions with shared transcriptional profiles. (**B**) Heatmap showing the top 8 differentially upregulated genes across Leiden clusters. The heatmap displays the scaled expression values, with red indicating high abundance and blue indicating low abundance within each cluster. (**C**) Gene Ontology Biological Process Pathway Enrichment results generated using EnrichR for each Leiden cluster. The top 5 pathways for each cluster are displayed based on their *p*-values, with red bars representing the pathways with the lowest *p*-values and blue bars representing the pathways with higher *p*-values. The gene counts in each pathway are shown along the x-axis.

Pathway enrichment analysis of Leiden clusters was undertaken (Supplementary Table S2). The table contains enriched GO terms, overlap ratios, adjusted *p*-values, odds ratios, combined scores, and contributing genes for each cluster. The spatial distribution of these clusters, as indicated by the colour-coded Leiden clusters on the H&E-stained organoid sections, is shown in Figure 1A. Cluster 1 exhibited a higher abundance of genes related to cell adhesion, such as LTF [19], which were more abundant compared to other clusters. Furthermore, this included cell migration genes such as PLAU [20] and TPM1 [21] (Figure 1C, Cluster 1). Notably, genes involved in the mitochondrial electron transport chain and cellular respiration, including MT-ND2 and NDUFA1, were lower in abundance in this cluster, contrasting with Cluster 3, where these genes were highly abundant (Figure 1B). This suggests potential metabolic differences between the identified clusters, with Cluster 3 displaying increased mitochondrial gene activity, which may indicate higher energy demand or oxidative metabolism (Figure 1C, Cluster 3). While high mitochondrial gene expression can also be associated with cell stress or apoptosis, in our dataset such regions exhibited variable cellular densities that complicate direct comparison of total transcript abundance; moreover, the Giotto workflow applies spot-level normalization to account for differences in sequencing depth. Histological assessment did not reveal apoptotic features. Cluster 2, represented by yellow spots, displayed a higher abundance of genes involved in protein catabolism and antigen processing, including members of the cathepsin protease family (CTSB, CTSS, and CTSD) (Figure 1B) in addition to MHC class II complex genes (HLA-DRA, HLA-DMB and HLA-DPA1) (Supplementary Table S1). These suggest an active role in proteolysis and immune response within this cluster [22]. Cluster 4 was primarily characterized by the elevated abundance of ribosome-associated genes, such as RPL21 and RPL29, which suggest active translation processes as shown in GO Biological Process Pathway enrichment (Figure 1C, Cluster 4).

**Figure 2:**
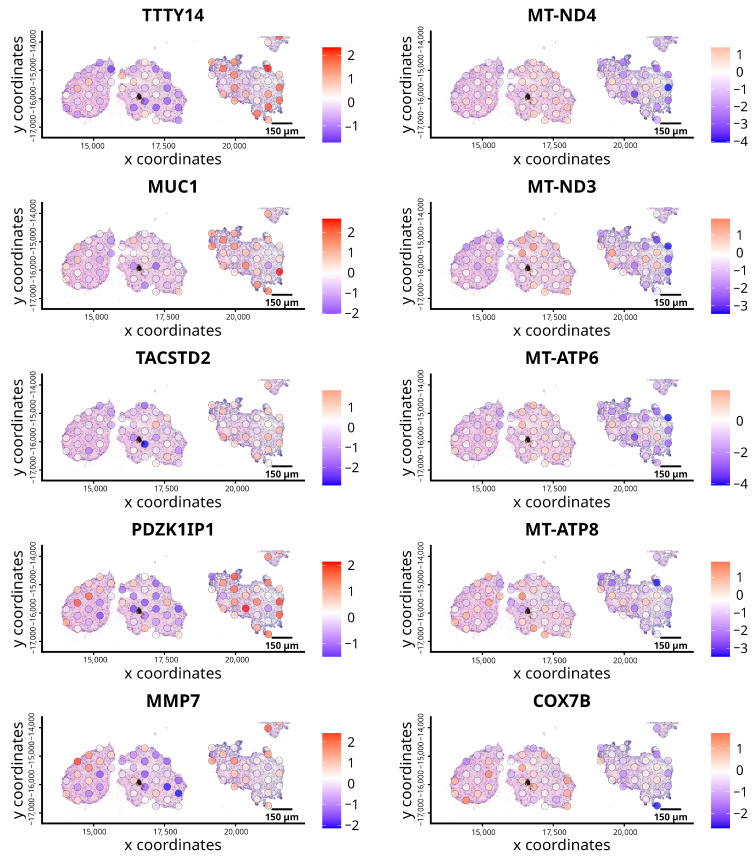
Spatial plots of expression patterns of top differentially expressed genes in control organoids identified by the scran method. Each panel represents the spatial distribution of gene expression for a specific gene across the organoid sections. The colour gradient, from blue to red, represents the relative expression levels, with red for higher expression and blue for lower abundance. The genes displayed include TTTY14, MUC1, TACSTD2, PDZK1IP1, MMP7, MT-ND4, MT-ND3, MT-ATP6, MT-ATP8, and COX7B, illustrating varying expression levels across spatial clusters.

Figure 2 shows the spatial distribution of the top differentially expressed genes as identified by the scran method, across the untreated organoid section. The expression of mitochondrial genes, including MT-ND4, MT-ND3, MT-ATP6, MT-AT8, and COX7B, was very variable; one organoid showed distinct expression around the edge, suggesting possible reduced mitochondrial activity. Mitochondrial metabolism is a common altered feature of ccRCC [23,24]. Furthermore, genes such as TACSTD2, MUC1, TTTY14, PCZK1IP1 and MMP7 appeared to show expression patterns converse to mitochondrial genes within the same organoid as evident in the normalized expression heatmap shown in Figure 1B.

### 3.2. Gene Isoform Expression in ccRCC Organoids

**Figure 3:**
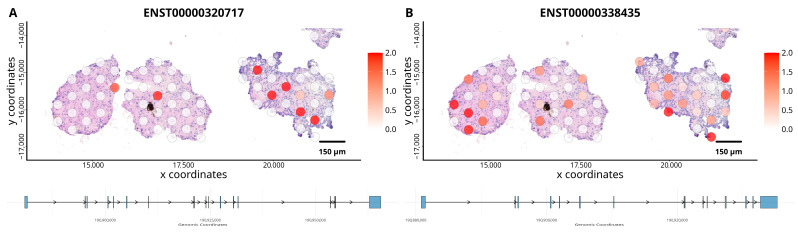
Spatial expression of GLS isoforms across ccRCC organoids. (**A**) Spatial distribution of the Glutaminase KGA isoform (ENST00000320717), and (**B**) GAC isoform (ENST00000338435). The colour scale, ranging from white (low expression) to red (high expression), indicates relative transcript abundance at the spatial coordinates of the organoids.

Analysis of differential isoform usage (DTU) showed altered abundance of GLS gene in organoids, the spatial expression patterns of the GAC isoform (Figure 3B), which encodes a more metabolically active form of the enzyme, shows that it is predominantly expressed in regions of the organoids with higher cellular density. The KGA isoform (Figure 3A), although present at lower overall levels, shows focal expression in specific areas of the organoids. Metabolism of glutamine is a key feature of ccRCC [25] and is a potential therapeutic target [26], glutaminase (GLS) has two main variants the KGA and GAC isoforms expression of which is implicated in cancer progression [27].

### 3.3. Treatment of Organoids with NUC-7738 Demonstrated Marked Changes in Spatial Gene Expression

Some organoids were treated with NUC-7738, which is in phase I/II clinical studies. Subsequently, the two datasets from control and treated organoids were integrated into a single object using the Giotto suite, allowing for direct comparison of spatial gene expression profiles between organoids. By applying the Giotto Suite for analysis, we normalized gene expression across organoid sections using its built-in Harmony algorithm [28] to account for RNA data batch variability. This correction was validated by comparing Leiden clustering and tissue contribution before and after integration (Supplementary Figure S6), confirming that batch effects were mitigated and enabling comparisons across sections and experimental runs, with observed differences reflecting treatment-specific changes while preserving spatial context.

**Figure 4:**
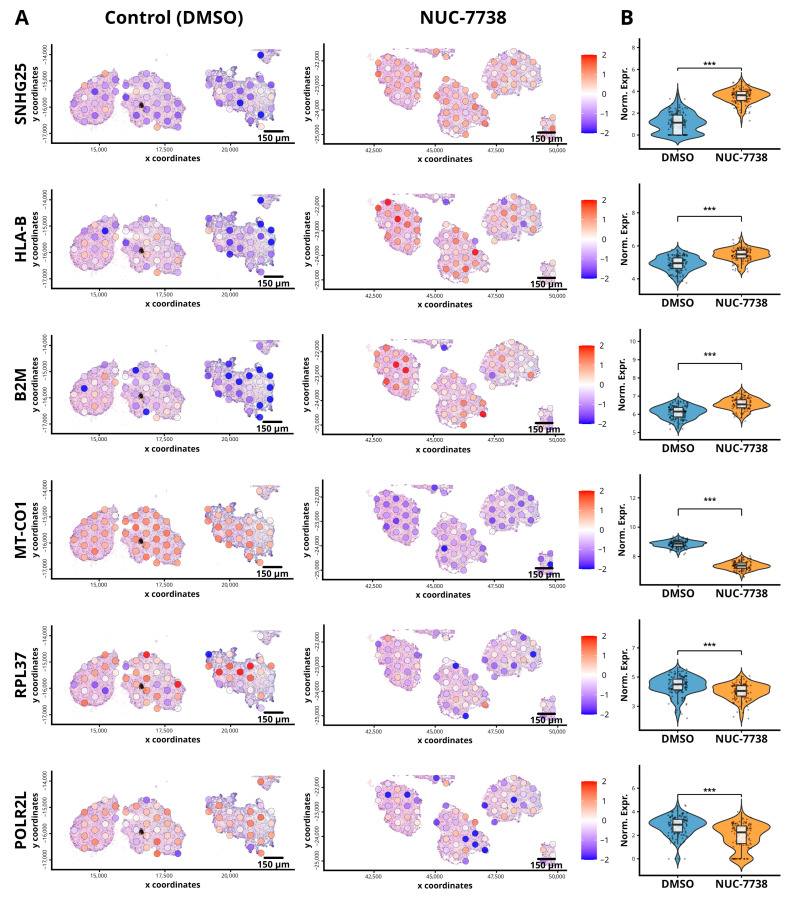
Differential Gene Expression and Spatial Distribution in ccRCC organoids after treatment with NUC-7738. (**A**) Spatial plots depicting the spatial distribution of the differentially expressed genes SNHG25, B2M, and HLA-B across organoids. The colour gradient, ranging from blue (low expression) to red (high expression), represents the relative abundance of these genes in specific spatial regions, highlighting their heterogeneous expression patterns following treatment with NUC-7738 (**B**) Violin plots with embedded box plots and individual points representing normalized expression across all detected spots in the section for each gene. Statistical significance was assessed using scran; *** (*p* < 1 × 10^−5^).

Differential expression analysis was performed on the merged Giotto object using a pseudobulk approach using scran method, and the resulting gene lists are presented in Supplementary Table S3. It lists the top genes per cluster, including raw and adjusted *p*-values (FDR), log fold change (logFC), gene name, cluster identity, and ranking. The results showed a significant increase in the abundance of genes, including the SNHG family of non-coding RNAs and immune-related genes such as HLA-B and B2M (Figure 4A,B). Spatial plots (Figure 4A) illustrate the heterogeneous distribution of these genes across organoids, while violin plots (Figure 4B) summarize expression levels across all detected spots. These findings align with our previous observations in biopsies taken from patients before and after treatment with NUC-7738 [29]. Additionally, after treatment with NUC-7738 we observed a lower abundance of mRNA from mitochondrial genes such as MT-CO1 and ribosomal subunit gene expression such RPL37, shown in Figure 4. While certain genes, including MT-CO1, SNHG25, and HLA-B, exhibited relatively uniform expression changes across organoids, others such as RPL37 displayed more spatially restricted patterns. These observations indicate that, although overall trends may be consistent, some regions show limited or no change, suggesting that treatment responses can be region-specific.

**Figure 5:**
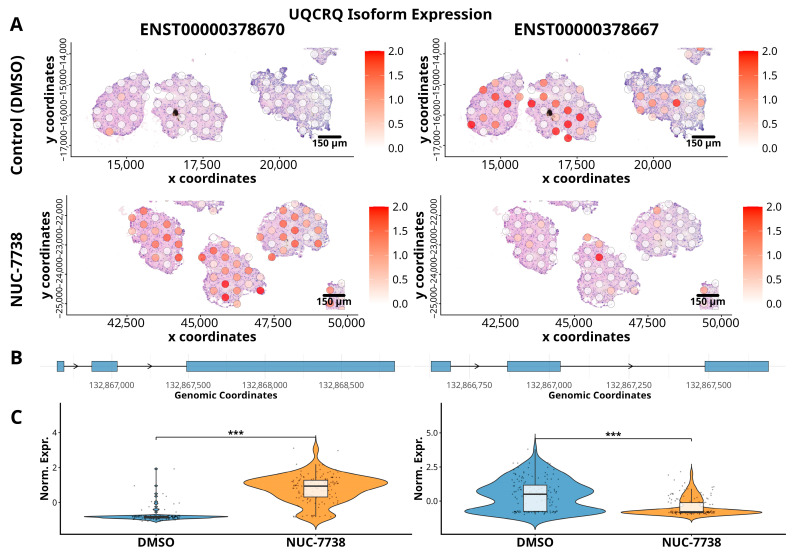
Spatial expression of UQCRQ gene isoforms in ccRCC organoids treated with DMSO (Control) and NUC-7738. (**A**) Spatial expression patterns of two UQCRQ gene isoforms, ENST00000378667 (left) and ENST00000378670 (right), across control untreated organoids and NUC-7738-treated organoids. (**B**) Isoform structures of the UQCRQ isoforms. Expression levels are colour-coded, with red indicating higher expression. The lower panel depicts the isoform structures. (**C**) Violin plots with overlaid box plots comparing normalized expression levels of each UQCRQ isoform between DMSO- and NUC-7738-treated organoids. Each data point represents expression from an individual spot across the entire section. Statistical significance was assessed using DRIMseq, with *** *p* < 1 × 10^−5^.

Furthermore, differential transcript usage (DTU) analysis between control and NUC-7738 treated organoids revealed changes in isoform usage for multiple genes (Supplementary Table S4), which lists gene and transcript IDs, likelihood ratios, degrees of freedom, *p*-values, adjusted *p*-values, and gene names. Among these, changes in UQCRQ were the most different. As illustrated in Figure 5, the ENST00000378667 isoform decreased, while the ENST00000378670 isoform increased upon treatment.

## 4. Discussion

Organoid models have shown promising avenues for recreating tumour heterogeneity [30,31] and being used for pre-clinical validation of novel drugs [32,33]. The greater use of organoids [34] has uncovered important aspects of cancer biology, such as transcriptome alternations in oxaliplatin resistance colorectal cancer [35] and enrichment of resistant subclones during treatment of pancreatic cancer [36]. Studies using the ccRCC organoid model are limited [37,38]. In this proof-of-principle study we demonstrate that patient-derived ccRCC organoids used before passaging, allow long read spatial transcriptome analysis of the organoid microenvironment before and after an experimental treatment.

Although spatial transcriptomics analysis of cancers is increasing [12,13,14], studies mainly employ FFPE and short-read RNA sequencing with the application of long-read analysis being limited [39]. The use of spatial transcriptomics in organoids has not been previously reported, in part probably due to the challenges of coping with the small size of organoids. However, in this study we have established ccRCC organoids and sectioned them onto 10× Genomic Visium slides, thus obtaining sufficient quality RNA to allow Nanopore v14 library construction. This increased the accuracy of RNA sequencing and basecalling compared with previous Nanopore chemistries.

To analyze the complex spatial transcriptomic data, we developed a bioinformatics pipeline that enabled integration of long-read sequencing data with spatial mapping using the Giotto suite, an open-source spatial analysis tool for multi-modal data [16]. Giotto enabled the creation of data objects from gene expression and transcript matrices generated by the wf-single-cell processing pipeline, allowing for data analysis through Leiden clustering to create spatial expression-informed clusters. In addition, visualization plots were produced to show gene expression superimposed on the H&E high-resolution images. The built-in scran method [17] for differential expression analysis facilitated the identification of gene expression differences among clusters, as well as the interactive selection of spatial regions for analysis. Our pipeline also accommodated multi-section analysis to be performed using the function joinGiottoObjects to analyze samples that were embedded on separate 10× Visium sections and sequenced independently, enabling normalization and analysis across sections.

By applying spatial transcriptomics, we mapped gene expression within and across organoids, preserving the architecture and revealing localized transcriptional variability. Spatial analysis coupled with long-read sequencing, uncovered multiple distinct transcriptomic profiles. This underscores the importance of examining tumours at a granular, spatially resolved level. Notably, differences in gene abundance related to proteolysis, mitochondrial function, and other metabolic pathways highlight the functional diversity underlying ccRCC heterogeneity. Given that ccRCC is marked by significant metabolic reprogramming [40,41] and its dependence on glutamine metabolism [42], our spatially resolved transcriptomic data provided insight into how individual organoid regions exploit specific metabolic routes. Using this approach, we further explored isoform-level diversity using DTU DRIMseq [18] analysis and found that the GLS (glutaminase) gene have variable expression of their isoforms GAC and KGA. Such insights pave the way for improved understanding of the tumour microenvironment, the complexity of alternative splicing, and the rational design of targeted therapies.

Furthermore, we evaluated the changes in the transcriptome in organoids treated with NUC-7738, a potent modifier of polyadenylation during transcription [15]. This showed significant changes in the expression of genes, for example, those involved in mitochondrial function, cellular metabolism and immune presentation; this replicated what was previously demonstrated using long read-RNA-seq in paired biopsies taken from patients before and after treatment with NUC-7738 [29]. Furthermore, spatial characterization revealed heterogeneity of response, potentially a very important consideration in assessing the ability of a novel treatment to efficiently target the entire tumour, rather than leaving unaffected subclones that ultimately may regrow.

The workflow paves the way for further development for single cell resolution spatial transcriptomics analysis, with drug treatment in addition to integration of other omics such as proteomics and metabolomics in the analysis of organoids. While this study was limited to 50 µm-resolution Visium and multiple organoids from a single patient sample, the core principle, using cryostat sections of tiny, non-passaged, patient-derived organoids for spatially resolved long-read transcriptomic analysis and treatment response profiling, is robust and scalable and resolution will be enhanced with next generation spatial technology.

## 5. Conclusions

This novel study demonstrates the successful application of long-read spatial transcriptomics to patient-derived clear cell renal cell carcinoma (ccRCC) organoids, enabling simultaneous analysis of spatial gene expression and transcript isoform variation within a physiologically relevant 3D model. The approach revealed pronounced regional heterogeneity in mitochondrial and ribosomal gene activity and identified spatially distinct patterns of glutaminase isoform expression. These findings underscore the metabolic and transcriptional complexity that exists within individual tumours and highlight the value of combining patient-derived models with spatially resolved transcriptomic technologies.

Treatment with the transcription-modifying agent NUC-7738 induced widespread transcriptional remodelling, including reduced expression of mitochondrial and ribosome-associated genes, reflecting its mechanism of action. Together, this workflow establishes a robust experimental platform for studying spatial transcriptional dynamics and treatment responses in cancer. The integration of long-read sequencing with spatial transcriptomics in organoid models offers new opportunities to dissect intra-tumour heterogeneity and to guide the development of targeted and personalized therapies.

## Data Availability

Raw Oxford Nanopore Visium v1 spatial transcriptomics reads from patient-derived ccRCC organoids (DMSO and NUC-7738 treated) are available in the Genome Sequence Archive (GSA-Human, accession HRA019526) at https://ngdc.cncb.ac.cn/gsa, (accessed on 19 December 2025). The complete analysis code and input data with step-by-step documentation is available at GitHub https://github.com/MustafaElshani/TumoroidSpatialLongRead, v0.0.2 (accessed on 3 January 2026).

## Supplementary Materials

The following supporting information can be downloaded at: https://www.mdpi.com/article/10.3390/cancers18020254/s1. Supplementary Figure S1: Whole-section spatial distribution and UMAP of Leiden clusters. Supplementary Figure S2: Whole-section gene expression maps corresponding to Figure 2. Supplementary Figure S3: Whole-section isoform expression maps corresponding to Figure 3. Supplementary Figure S4: Whole-section feature expression maps corresponding to Figure 4. Supplementary Figure S5: Whole-section feature expression maps corresponding to Figure 5. Supplementary Figure S6: Batch effect assessment before and after Harmony integration. Supplementary Table S1: Top differentially expressed genes defining Leiden clusters (DMSO condition). Supplementary Table S2: Enrichr-based Gene Ontology Biological Process enrichment of cluster-specific genes. Supplementary Table S3: Differential gene expression results from pseudobulk analysis using scran (DMSO vs 30 µM NUC-7738). Supplementary Table S4: Differential transcript usage analysis using DRIMSeq comparing untreated and NUC-7738-treated tumoroids.
