# Supplementary material for: Long-Read Spatial Transcriptomics of Patient-Derived Clear Cell Renal Cell Carcinoma Organoids Identifies Heterogeneity and Transcriptional Remodelling Following NUC-7738 Treatment
Source: Cancers (Basel). 2026 Jan 14;18(2):254. doi: 10.3390/cancers18020254 (PMC12839147; doi:10.3390/cancers18020254)
Supplement: Supplementary file 1 [file cancers-18-00254-s001.zip › Supplemntary_Figures.pdf]

Long-read spatial transcriptomics of patient-derived clear cell renal cell carcinoma organoids identifies heterogeneity and transcriptional remodelling following NUC-7738 treatment

Supplementary Information

Supplementary Figures

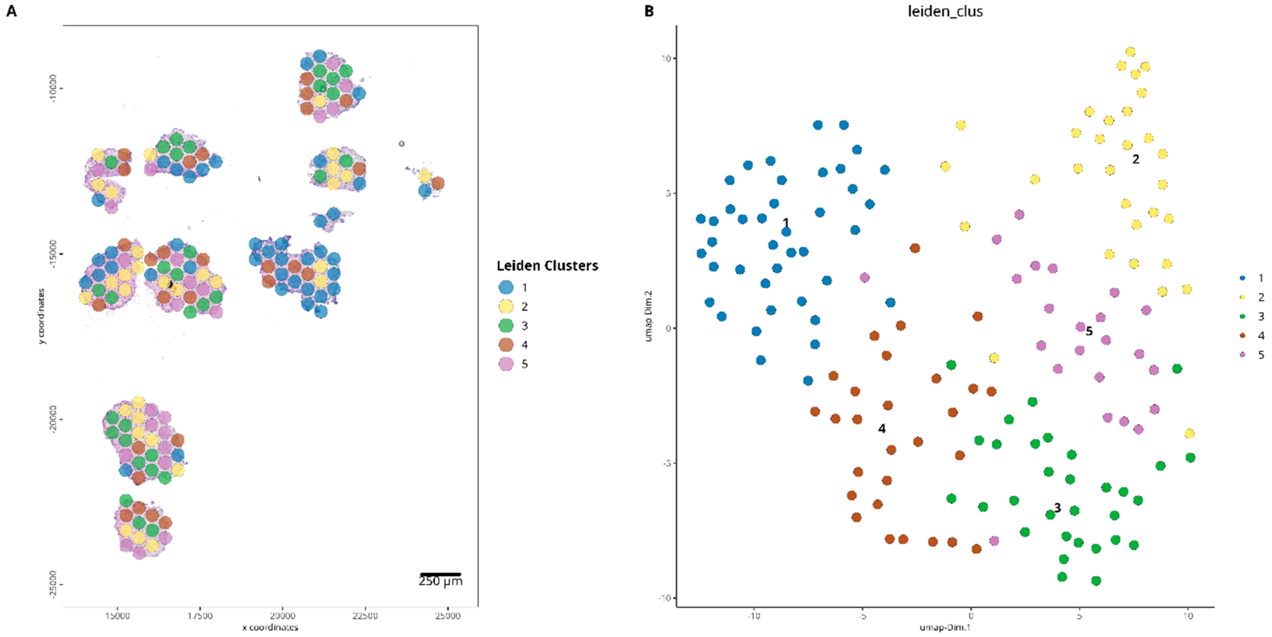

Supplementary Figure S1. Whole-section spatial distribution and dimensionality reduction of Leiden clusters.

(A) Spatial map of the entire Visium section showing the spatial localisation of spots assigned to Leiden clusters (1–5), overlaid on the tissue image. Each colour represents a distinct Leiden cluster.

(B) UMAP projection of the same spots, coloured by their Leiden cluster membership, illustrating transcriptional similarity between spots independent of their spatial location.

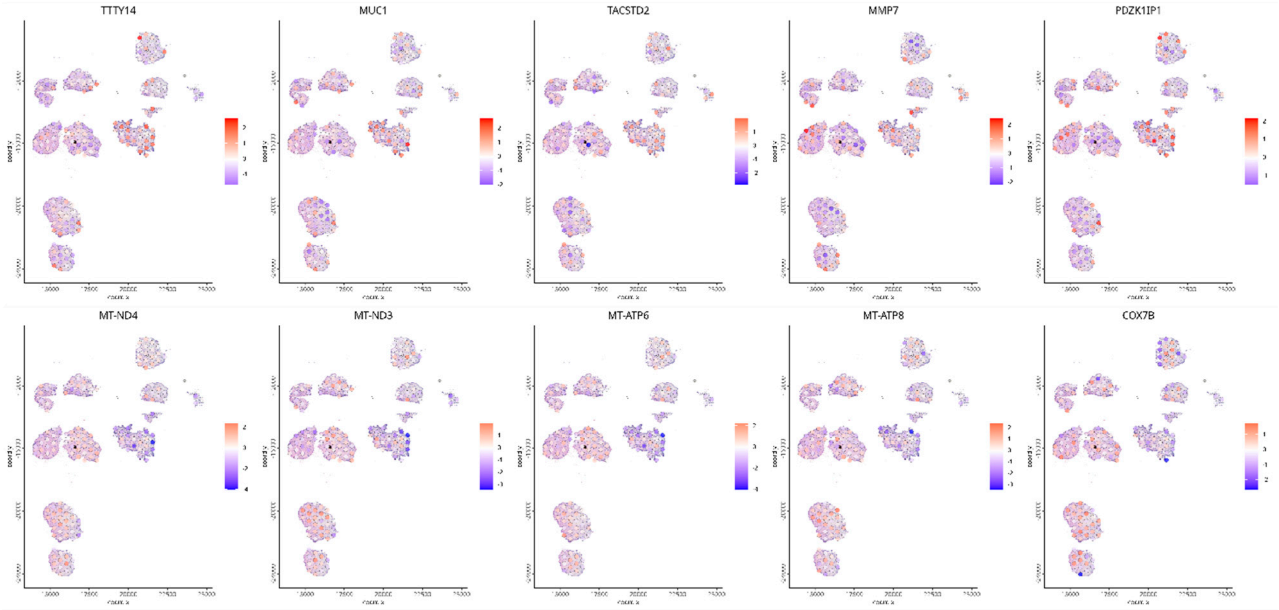

Supplementary Figure S2. Whole-section representation of gene expression patterns shown in Figure 2.

Spatial maps displaying expression of the top differentially expressed genes across the entire tissue section, corresponding to the genes presented in Figure 2.

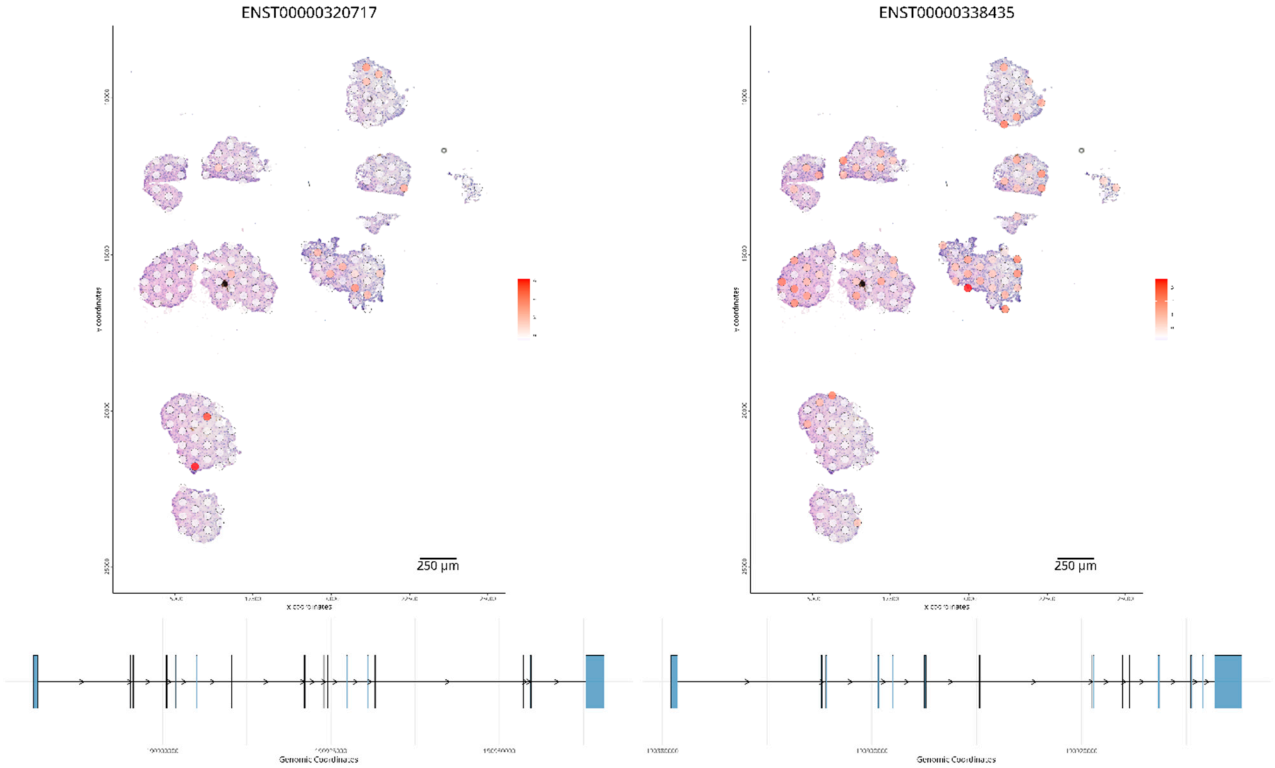

Supplementary Figure S3. Whole-section representation of Figure 3.

Spatial maps showing expression patterns for the isoforms presented in Figure 3 across the entire tissue section.

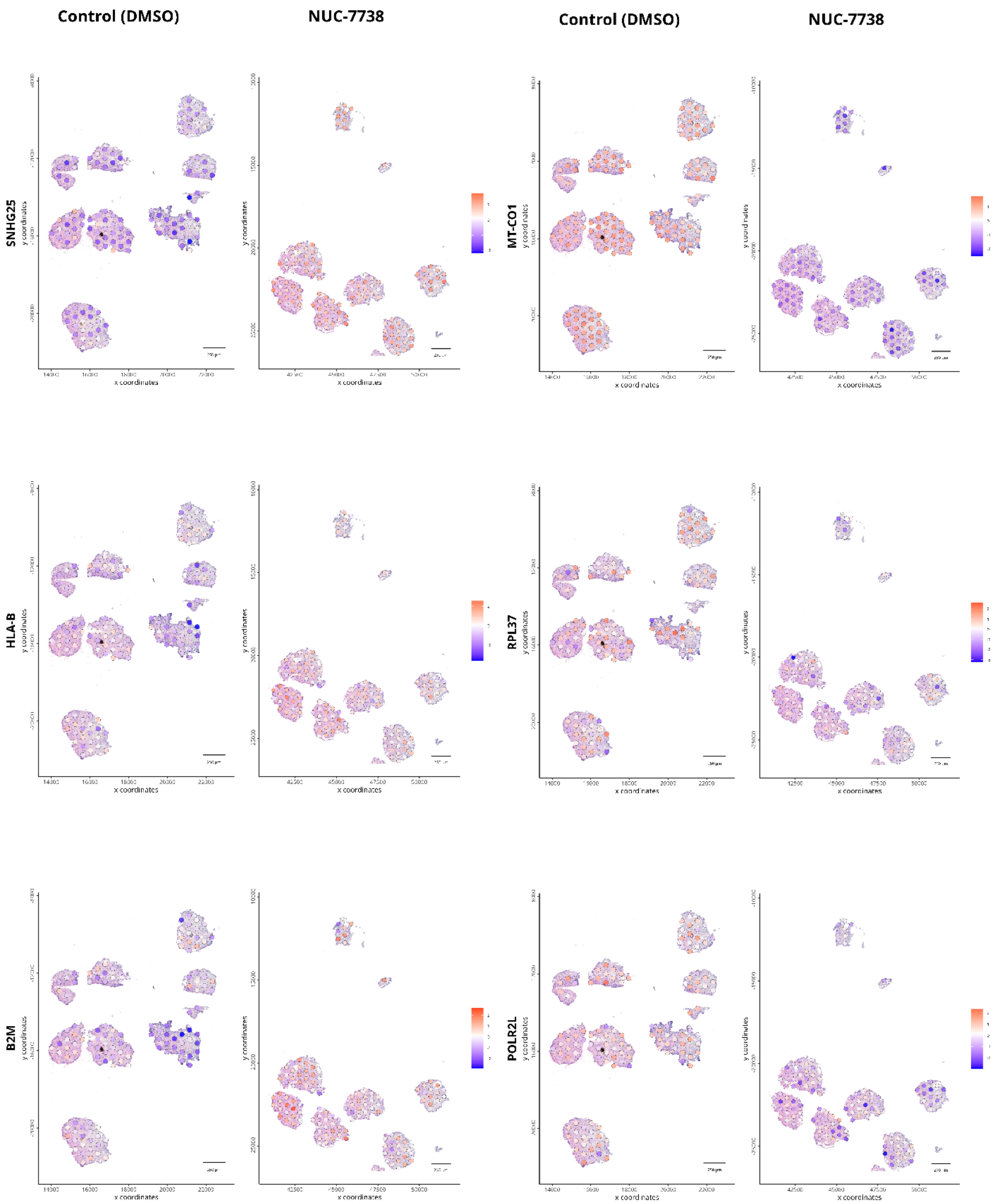

Supplementary Figure S4. Whole-section representation of Figure 4.

Spatial maps showing expression patterns for the features presented in Figure 4 across the entire tissue section.

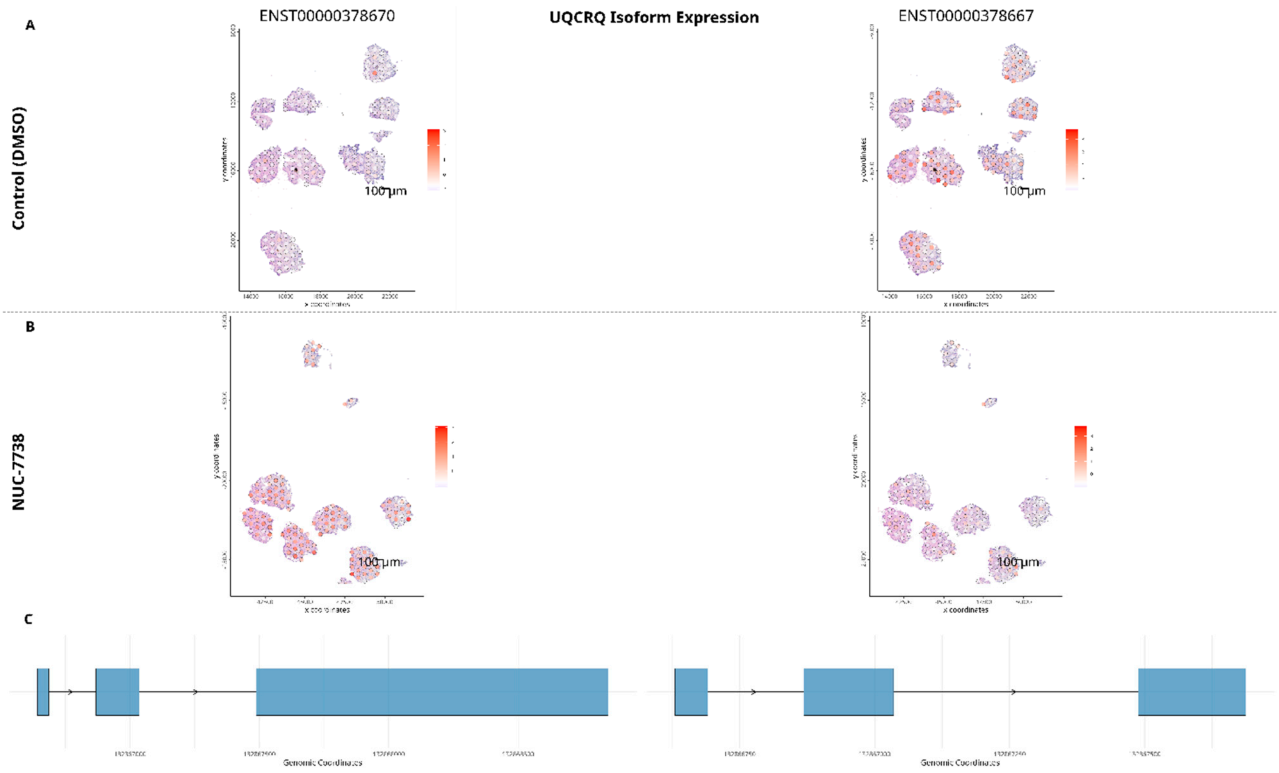

Supplementary Figure S5. Whole-section representation of Figure 5.

Spatial maps showing expression patterns for the features presented in Figure 5 across the entire tissue section.

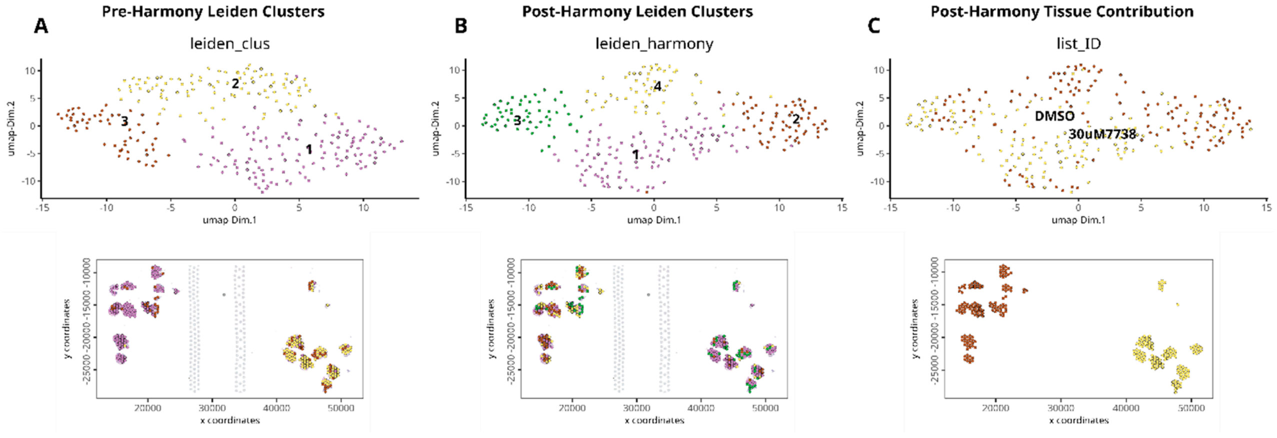

Supplementary Figure S6. Assessment of batch effects before and after Harmony integration.

(A) Pre-Harmony Leiden clustering showing that clusters were largely segregated by tissue section (DMSO vs NUC-7738 treated), indicating potential batch effects.

(B) Post-Harmony Leiden clustering after integration, demonstrating more even mixing of cells from both sections across clusters, suggesting successful batch correction.
(C) Post-Harmony tissue contribution visualizing the origin (condition) of each spot in UMAP space, confirming balanced representation from both DMSO and NUC-7738 conditions across clusters.

Supplementary Tables (.xlsx)

Supplementary Table S1: Leiden clusters identified from Giotto analysis, representing distinct gene expression profiles (five clusters in total).
Supplementary Table S2: Enriched Gene Ontology (GO) Biological Process terms derived from cluster-specific genes.
Supplementary Table S3: Differential gene expression results from pseudobulk analysis using the scran method, including logFC, raw and Holm-corrected p-values, and cluster identities.
Supplementary Table S4: Differential transcript usage (DTU) results from DRIMSeq analysis, reporting changes in isoform usage between control and NUC-7738-treated tumoroids.
